# Nursing Professionals’ Perceptions of Career Planning and Development in Nursing and Organisational Support

**DOI:** 10.1155/jonm/8843697

**Published:** 2026-03-18

**Authors:** Hanna Kallio, Marita Koivunen, Hanna Liljeroos, Anne Kuusisto, Mari Kangasniemi

**Affiliations:** ^1^ Department of Nursing Science, Faculty of Medicine, University of Turku, Turku, Finland, utu.fi; ^2^ The Wellbeing Services County of Satakunta, Satakunnan Hyvinvointialue, Pori, Finland; ^3^ Research and Development Unit, The Wellbeing Services County of Satakunta, Satakunnan Hyvinvointialue, Pori, Finland; ^4^ Varsinais-Suomen Hyvinvointialue, The Wellbeing Services County of Southwest Finland, Turku University Hospital, Turku, Finland, vsshp.fi

**Keywords:** career planning, development, managerial roles, MeSH: career planning, nurse managers, nurses, organisational perspective, organisational structures, qualitative research

## Abstract

**Background:**

The global shortage of nursing staff has drawn attention to the limited appeal of a career in nursing. Career planning and development for nurses highlights the importance of individual career interests but also the role of nurse managers in facilitating career opportunities.

**Aim:**

To describe nurses’ and nurse managers’ perceptions of career planning and development in nursing. This knowledge is needed to support nurses and develop sustainable workforce strategies for nursing management.

**Methods:**

A qualitative descriptive study was conducted using semistructured interviews and analysed through inductive content analysis. Data were collected in 2022 from 26 nurses and nurse managers at one central hospital and two healthcare centres in Finland. The semistructured interview guide was developed based on previous literature.

**Results:**

Based on our results, two main categories emerged. Firstly, career planning and development in nursing was defined as a goal‐oriented professional strategy to which nurses have a professional right. This was seen as a means of expanding nurses’ competencies, enabling them to take on advanced roles, and strengthening their influence in care across all career paths from bedside nursing to leadership positions. Secondly, organisational support for nurses’ career planning and development was considered essential and should be multilayered and progressive. Key elements included career‐oriented organisational values, a clear and achievable career model within the organisation itself and consistent daily support and follow‐up mechanisms.

**Conclusions:**

Career planning and development addresses nurses’ need for personal and professional development. Nurse managers can facilitate this by facilitating peer mentoring and a career‐oriented work culture, expanding career opportunities beyond the unit level and developing career ladders as a part of sustainable workforce planning to improve nurses’ retention in the profession. Further evaluative and comparative research on this topic is needed.

**Summary**
• What is already known?
◦ Poor career opportunities weaken the position of nurses in working life and hinder the attractiveness of and retention in nursing.
◦ Organisational structures play key role in providing nurses with equal opportunities for career planning and development.
• What does this paper add?
◦ This paper shows how nurses and nurse managers in Finland perceive career planning and development and what is required for their organisations to support it.
◦ The paper identifies the key role and challenges of nurse managers in supporting nurses’ career planning and development.

## 1. Introduction

Nurses appreciate having appropriate challenges at different phases of their careers [1, 2]. As a concept, career planning and development refer to an intentional process [3] through which individuals pursue professional growth and meaningful challenges in their working lives [4–6]. This process builds on an individual’s educational achievements and continuous development of competencies [7]. Successful career development has been shown to enhance nurses’ motivation, engagement [8] and their sense of meaningfulness [1].

However, nurses report that opportunities for recognition and career advancement are limited [9] and that role expectations are often unclear and confusing [10, 11]. Traditionally, career development opportunities in nursing have focused on leadership, research and development [2] and teaching [12], while clinical career opportunities have focused on different specialisms at the advanced nursing level [13]. Less attention has been given to career opportunities within direct care [9, 14, 15]. Nurses’ own initiatives [9], as well as their levels of interest, motivation [16] and communication skills [17] play a central role in pursuing career development. Moreover, career development is associated with increased employee effort [18].

At an organisational level, nurse managers and the structural frameworks within organisations play a crucial role in nurses’ career planning and development [19]. Frontline nurse managers are key actors in aligning nurses’ competencies with suitable tasks [16] and in supporting career progression at the unit level [16, 20]. However, nurse managers have expressed the need for clear criteria and practical tools to support this process [6]. They have also reported a lack of organisational support [21] and unclear expectations when transitioning into leadership roles [22]. Therefore, transparent and accessible organisational structures that offer all staff equal opportunities for career advancement are essential [5]. These structures should enable progression to be more predictable than haphazard [21]. Clear and open supporting policies have been identified as a key requirement for competence and career progression among nurses [14].

The global shortage of nursing staff, coupled with the limited international appeal of nursing as a career, is also evident in Finland [14, 19, 23]. The limited appeal has reduced nurses’ interest in developing their competencies [16, 17] and increased staff turnover, resulting in decreased interest in the care sector among future generations [2, 24, 25]. Many nurses enter the profession with a desire to help others, but they can become frustrated when poor career opportunities and working conditions prevent them for achieving this goal [20]. It is notable that a lack of career advancement opportunities also compromises care delivery [3] and the overall quality of services [16, 17]. Although initiatives such as career ladders and a national framework for advanced nursing expertise [27, 28] have supported nurses’ careers in Finland, little is known internationally about nurses’ career planning and development in their daily work within the organisations. Thus, the aim of this study is to describe nurses’ and nurse managers’ perceptions of career planning and development in nursing. This knowledge is needed to support nurses in their career paths and enable nursing management to develop and implement [24] sustainable workforce strategies for nurses’ career planning and development. Although this study was conducted in Finland, nurses’ career planning and development remain underexplored internationally, highlighting the broader relevance of these findings for nursing management practice.

The research questions are as follows:

1. How do nurses and nurse managers characterise nurses’ career planning and development?
2. How can healthcare organisations support nurses’ career planning and development?

## 2. Materials and Methods

### 2.1. Study Design

We used a qualitative descriptive design [29] involving semistructured [30] individual and group interviews with nurses and nurse managers in Finland. The research was conducted in 2022. The data were analysed using inductive content analysis [31]. The study was reported in line with the Consolidated Criteria for Reporting Qualitative Research (COREQ) checklist [32] (Supporting file 1).

### 2.2. Participants and Recruitment

We used a purposive sampling strategy to ensure that the participants had the necessary knowledge to enable them to respond to the aims of our study. To ensure maximal variation of the participants, we only used the most necessary inclusion criteria. Participants were eligible to enrol in this study if they: (i) volunteered, (ii) held a nursing degree and (iii) were employed as nurses or nurse managers in the participating hospital or in one of the two healthcare centres. The most important thing was that the participants felt they would contribute, which is why we did not restrict working experience. We openly informed about the study openly and recruited participants from all departments and units in one central hospital and two healthcare centres operating within one hospital district and primary healthcare services in Finland. We used two recruitment strategies [29] with the same inclusion criteria. First, a researcher (HK) informed contact persons in these, who then delivered an information letter to nurses and nurse managers. This letter included a description of the purpose and process of the study, assurance that participation would be voluntary and confidential, contact details for the researchers, and a link to the interview guide. The second strategy was snowball sampling [29] in which interviewees were asked to encourage their colleagues to participate. We continued recruitment until no new volunteers were enrolled. In total, 26 voluntary participants enrolled in the study, of whom 19 were nurses working in various clinical roles (Table 1) and the rest were nurse managers who worked as supervisors. In terms of education, most of the participants had a master’s degree or equivalent, all but one was female, and their mean age was 48 years (see Table 1).

**TABLE 1:** Participants’ (*n* = 26) background information.

	***n***

Age	
27–63 years (Average: 48)	
−29	1
30–34	0
35–39	4
40–44	7
45–49	1
50–54	5
55–59	5
60–	3
Gender	
Female	25
Male	1
Work setting	
Hospital	15
Nurse Mangers	*4*
Nurses	*11*
Primary healthcare services	11
Nurse Managers	*3*
Nurses	*8*
Professional role/title	
Nurse Managers	7
Ward Manger	*6*
Service Manager	*1*
Nurses	19
Registered Nurse	*10*
Nurse Coordinator or Instructor	*3*
Clinical Nurse Specialist	*2*
Clinical teacher	*1*
Paramedic	*2*
Dental hygienist	*1*
Highest educational degree	
Doctoral degree in Health Sciences	1
Master’s degree in Health Sciences	1
Master’s degree in Nursing	9
Master’s degree in Social Work	2
Bachelor’s degree in Nursing	11
Vocational degree in Nursing	2

### 2.3. Data Collection

We used semistructured interviews to collect the data and allow participants to describe their perceptions freely while remaining focused on the study topic [30]. We conducted a literature review [14] to develop an interview guide with four main questions and follow‐up questions. Question 1 explored the meaning of the career and career planning in nursing, including career paths, progression and characteristics of a good career. Question 2 focused on how career planning is addressed in nursing education and working life, and question 3 focused on the significance of career from the perspective of individual nurses, the profession, organisations and society. Question 4 addressed the means and implementation of career planning, highlighting the roles of nurses, managers, organisations and societal structures (Supporting Table 2). The interview guide was pilot‐tested with one nurse and one nurse manager and found to be comprehensive and understandable. Thus, there was no need to amend it, and the pilot interviews were included in the research data.

The interviews were conducted with individual participants (*n* = 19) and groups (*n* = 2) of three or four participants, online (Zoom) or as face‐to‐face meetings, between September and November of 2022. The interviews were conducted by three researchers (HK, MKO, MKA) who have experience of both nursing and qualitative research interviews and who were previously unknown to the interviewees. All interviews were audio recorded, and their duration varied from 33 to 78 min (42 min on average), amounting to 14 h 35 min in total. Although we recognised that data saturation had been reached when no new themes or insights emerged [29], three additional interviews were conducted to ensure credibility and saturation of the study.

### 2.4. Data Analysis

We analysed the data using inductive content analysis [31] with the help of NVivo Version 12 software. First, the interviews were transcribed verbatim, yielding 302 pages of text (Times size 12, line spacing 1.5). After that, all the manifest expressions that answered our two research questions were extracted from the data, and those referring to the same content were grouped together. These groups were then abstracted and categorised based on their similarities and differences. Finally, the categories were named inductively to describe and encompass the underlying content [31]. From this, we identified eight subcategories and two main categories. The initial extraction and grouping of relevant expressions was carried out by one researcher (HK) with strong expertise in qualitative methods. The abstraction, grouping and naming of the categories were then developed and finalised by the research group, which included members with backgrounds in clinical nursing, nursing management, ethics, and qualitative research. This multiagency approach helped us to minimise interpretation bias and reduce the influence of individual researchers.

### 2.5. Ethics

We applied for and received organisational research approval from the hospital district (Decision 49/2022) and primary healthcare services (Decision 170,822) before data collection. When assessing a study for approval, organisations in Finland evaluate its aim and quality, the need for ethical review, data protection measures and its impact on daily operations. According to Finnish legislation, this type of study with legally competent adult volunteers does not need ethical approval from an ethical committee [33]. However, we followed the national [34] and international principles of good research practices [35]. Based on the principles, we obtained informed consent from all participants and provided them with information about the aims of the study, confidentiality, the voluntary nature of participating, and their right to withdraw without consequences. Due to the limited number of participants, we did not collect or report detailed background information, and we reported the study in such a way that no organisation or individual participant could have been recognised.

## 3. Results

Based on our results, participants characterised career planning and development in nursing as an individual strategy to achieve professional goals and felt that multilayered, progressive organisational structures are required to support career planning and development (see Table 2).

**TABLE 2:** Results.

Main categories	Subcategories	Groupings
Nurses’ career planning and development as a goal‐oriented professional strategy	As nurses’ professional right	• Right to professional growth • Right to career advancement opportunities • Right to relevant training and competences
	Individualised target‐oriented professional progress	• Structured goal setting • Future‐oriented career development • Personal awareness and motivation
	Deepening of competencies and professionalism	• Development of knowledge and skills • Strengthening of professional identity
	Enables nurses to take on advanced duties at work	• Changes in job description • Expanded responsibilities • Independent decision‐making and practice
	Strengthening nurses’ roles as influencers	• Advancing nursing development • Influence over own work • Impact on work community

Organisational support for nurses’ career planning and development: a multilayered, progressive structure	Career‐oriented organisational values	• Strategic value recognition • Mutual nurse‐organisation benefits • Competent leadership • Career‐oriented work environment • Multilevel collaboration
	Clear and attainable career model in organisation	• Competence based role alignment • Career ladders • Transparent recognition and rewards
	Daily support and follow‐up	• Individual career planning • Reflection and performance reviews • Peer‐support and mentoring • Career development evaluation

*Note:* Categories and subcategories were developed inductively from coded interview data.

### 3.1. Nurses’ Career Planning and Development as a Goal‐Oriented Professional Strategy

Participants described career planning and development in nursing as (i) a professional right, (ii) driven by individual pursuit of professional goals, (iii) through which competencies and professional skills are deepened and (iv) nurses are enabled to take on higher‐level duties in their work. They also felt that (v) career planning and development enables nurses to exert influence over their work and in the healthcare industry (see Table 2).

#### 3.1.1. Nurse’s Professional Right

Participants described nurses’ career planning and development as a professional right. They said that all nurses should have the *right to develop* their professional expertise in order to provide high‐quality care and experience their working life as meaningful. The right to career growth opportunities in the field of nursing and clinical work was seen as equivalent to that in other industries. Therefore, nurses were seen to have the *right to career advancement opportunities* in their organisation. Participants called for new rights as employees to identify and create professional development opportunities in their organisation.

“*(In career growth) one’s job description changes* … *and becomes more meaningful* … *Today I widely know the city’s services and I can now better guide clients to proper services.”* (Participant 12; Registered nurse working as a service coordinator)

*“People are quite unaware of the (career) possibilities* … *different possibilities should be presented in the recruitment already.”* (Participant 10; Nurse manager)

Participants said that career planning and development incorporates their *right to relevant training and competencies.* They said that nurses should be able to work according to their competencies and career plans and receive appropriate further education and training to support them in performing their work tasks. Participants expected employers to support nurses in pursuing such education through flexible employment contracts and working arrangements. They highlighted the inevitable costs of pursuing further education and called for employers to contribute to meeting these where that education would ultimately benefit the organisation.

“*I know people who attend a higher university of applied sciences degree at their own expense and use days off and take unpaid days for those school days, but the employer gets the benefit of it then when they work as nurse managers*… *it eats motivation for their career path and self-development.”* (Participant 13; Registered nurse)

#### 3.1.2. Individualised Target‐Oriented Professional Progress

Participants perceived that career planning and development in nursing is primarily about *structured goal setting towards future-oriented career development*. It was seen as being driven by nurses’ individual preferences and targets and perceived as a pathway or journey towards achieving one’s own professional goals.

*“Career planning is a longer-term idea of how you want to follow your career path* … *It would be a scary thing if someone else planned my career for me. It has to be about what I want.”* (Participant 3; Paramedic)

Participants underlined the individual’s role in career planning and development, based on their *personal awareness and motivation.* They felt that it was important for a nurse to be aware of their own interests, understand their own developmental needs, and have a proactive attitude, for instance, taking the initiative to find out about training opportunities, plan for these, and raise them with the nurse manager. At the same time, any such desires to progress also need to be justified from the organisational perspective in terms of how further training would benefit the organisation. Participants noted that sometimes nurses lack the courage to change, and sometimes they become a novice again when they move to a new job. They also elaborated on a lack of career thinking and career competencies in the field of nursing and how this prevents nurses from setting career plans and goals. Nurse managers emphasised that career development requires targeted education. Suitability for such study also depends on their private life situation.

“*I want to get something new, learn something new and get to test my limits, develop.*” (Participant 18; Registered nurse)

#### 3.1.3. Deepening Competencies and Professionalism in Nursing

Participants felt that a crucial characteristic of career planning and development in nursing is the need to *advance their knowledge, skills, and competencies* and gain wider perspectives to meet changing requirements in their working lives. For instance, they highlighted the need to develop specialist skills and expertise in particular areas of nursing such as oncology or wound care. They associated career planning and development with the accumulation of work experience. They referred to ‘old hand’ nurses who had built competence through their work and gained respect, some working on the same ward for decades. Often, these experienced nurses substituted for nurse managers or acted as assistant nurse managers. In public healthcare in Finland, working years are tied to salary increases. Participants felt that it was wise for organisations to exploit nurses’ existing competencies and share these in the workplace. In this way, the competence of the whole unit or organisation improves. Career planning and development was associated with the *strengthening of professional identity*. It strengthened a person’s experience of professional significance, dignity, respect and pride.

“*In career development a nurse′s job description may shrink but skills expand*.” (Participant 11; Nurse manager)

*“In the work community, it is known that these nurses know a lot about this matter and others can turn to them.”* (Participant 10; Nurse manager)

“*Then I experience being respected, my training and experience being respected, when it shows up in my salary as well.”* (Participant 4; Registered nurse)

#### 3.1.4. Enabling Nurses to Take on Advanced Duties at Work

Participants described that career development manifested as c*hanges in the job description and content*. Various specialities and ambulatory nursing provided nurses different content of work. Also, career paths that are more indirectly related to care have been introduced, such as leadership, teaching, research, clinical nurse specialist, and project and developmental work. These are also related to *expanded responsibilities*. Participants characterised career development as a positive increase in new or updated duties and responsibilities at work. Nurse managers mentioned the opportunity for nurses to move into the role of a ‘nurse in charge’ which refers to the most experienced nurses who take the lead, coordinate, and organise tasks during a shift. They also said that career development could involve an increase in the number of units in which a nurse operated, possibly not just on one campus but also across a region.

“*You get more responsibility.”* (Participant 16; Dental hygienist)

Participants associated advanced duties with nurses having *increased independence in decision-making and practice*. Specific examples mentioned in this respect included pharmacological work, medical operations, and service delivery. Moreover, career development could involve a change in workplace, to other healthcare organisations, or even a move into new industries such as entrepreneurship, software development for patient care and selling health products.

*“We get training and authorisation to perform some operations or give medicines independently.”* (Participant 8; Paramedic)

#### 3.1.5. Strengthening Nurses’ Role as Influencers

According to participants, career planning and development contribute to *advancing nursing development*. They explained that nurses working in basic roles feel that their suggestions are rarely heard, but those with further education and career development have more power to apply new knowledge for the benefit of patients. They experienced this as rewarding.

*“I had so many developmental ideas, that weren′t heard (in basic clinical position) and I got bored to the point that it drove me (towards career development).”* (Participant 4; Clinical nurse specialist)

Participants linked career planning and development with nurses having better opportunities to have *influence over their own work*. Skilled nurses often experienced frustration when they had not been able to provide high‐quality care, but training gave them tools for addressing this. Nurses who developed their careers and competencies had more *impact in their work communities* because they acted as expert advisers in care practices or played leadership roles.

*“Yes, it rewards the nurse if one has studied certain things, developed oneself, and then is able to bring them into practical nursing tasks*… *Being able to use what [they’ve] learned and seeing that benefit the patient.”* (Participant 10; Nurse manager)

“*I have developed myself* … *and achieved such a role that* … *I can require nurses to act in a certain way*.” (Participant 10; Nurse manager)

### 3.2. Organisational Support for Career Planning and Development: A Multilayered, Progressive Structure

Our analysis suggested that career planning and development in nursing requires organisational support at many levels, including organisational values that support career progression, a clear and attainable model for pursuing it and daily support and follow‐up to support it (see Table 2).

#### 3.2.1. Career‐Oriented Organisational Values

In terms of integrating career planning and development into employers’ *strategic values*, participants emphasised the importance of the organisation offering equal, transparent and concrete but voluntary opportunities. Nurse managers called for incorporating it into organisational strategies and resourcing. Participants highlighted the importance of positive communication about career planning and development in organisations and the employer having a positive stance towards staff education. They brought up the significance of permanent work contracts in relation to career development, stating that substitute workers had to focus on the continuation of their employment contract at the expense of being able to pursue their career goals.

“*There should be some opportunity for everyone. Not so that two or three out of ten nurses have special expertise and attend training, but no one else can go. Or if they can, it is two hours here in the staff′s (break) room*.” (Participant 21; Registered nurse)

Participants recognised the *mutual nurse-organisation benefits* of career planning and development for both nurses and their organisations. They said that it helped individuals to feel better appreciated and that their work was meaningful. In turn, this strengthened their commitment to their work. They also felt that, as career planning and development was closely associated with competence development, it promotes quality of care and services. However, despite this, participants felt that their career development in nursing had been poorly supported to date. They brought up situations in which their career changes and promotions had come about through random or ‘lucky’ occurrences or through drifting. They said that learning institutions do offer training for nursing staff, but, without efforts on the part of employers, gaining further education does not automatically lead to gaining a new or better position at work.

*“Employers and nurse managers should be participating in nurses′ career planning*… *in the care industry*… *even if you educate yourself, you continue the same job with the same salary and nothing will change.”* (Participant 4; Registered nurse)

Organisations’ strategic values were felt to impact the resources needed for career planning and development, and resource scarcity was noted as a barrier to supporting nurses’ career development. If an employee is permitted to pursue training or take on new duties, their previous workload must be carried by someone else, and it can be difficult and expensive to resource this. COVID‐19 also challenged nurses’ daily work and opportunities for training. Nurse managers described that, from their point of view, the lack of resources prevented them from supporting nurses’ career planning and development. When a nurse leaves the unit, it is hard to find new employees due to labour shortages. Nurse managers also had limited time for career promotion activities. Overall, nurse managers’ influence over nurses’ career planning and development was considered to be significant.

*“I know units where employees haven’t told [the nurse manager] that they applied for some training because [they] won’t receive it well. … When it’s hard to get employees, the nurse manager won’t let that nurse go. Nurse managers have a lot of power to be an obstacle to nurses’ career planning.”* (Participant 1; Nurse manager)

Participants said that *competent nurse leadership,* willingness, and interest with regards to nurses′ career development influenced nurses’ opportunities to progress in their careers. Nurse managers need to be aware of career possibilities in nursing and, according to nurses, it is important that they knew their staff and understand their work. For this reason, distant leadership was felt to be problematic.

*“[The] arrangement of activities and development of job descriptions are very dependent on the nurse manager, [and] how eager they are to get involved in these things.”* (Participant 16; Dental hygienist)

Participants highlighted the significance of a *career-oriented work environment* that supports nurses’ career aspirations. They said that nurse managers play a key role in promoting an atmosphere amongst staff that is positive towards career planning and development. They could, for example, pass on information to their staff about career opportunities, get staff excited about new career opportunities in the organisation, promote positive attitudes towards colleagues’ studies, and ensure that new employees are comfortable and engaged in the unit.

*“My supervisor knew that I was studying for a master′s degree and said, in front of everyone, that she would never study nursing science. And everyone agreed that yes, nursing science is that kind of stupid occult science* … *I know people who haven′t told anyone (in their work community about their studies) because they have been afraid of other′s opinions.”* (Participant 7; Nurse manager)

Nurses’ career planning and development require *multilevel collaboration in the organisation*. Participants emphasised that career planning and development is a joint endeavour between nurses and the organisation, with common aims and mutual benefits. They argued that developing structures for career planning and development requires multiprofessional, multidisciplinary, and mutual collaboration. In this context, multiprofessional referred to the involvement of administrators, human resource management and recruitment, nurse managers, and staff representation from each group of professionals in the organisation. Multidisciplinary and mutual work emerged in relation to collaboration between healthcare, social services, and other healthcare specialities to support career development over sectoral boundaries. Networking at regional and national levels was also highlighted, for example, to learn from the career planning and development innovations of other organisations. Participants particularly focused on collaboration with educational institutions where nursing students could benefit from learning about career opportunities from nursing staff. Nurse managers were particularly keen to utilise the findings from nursing students’ theses and research in the workplace. Referring to collaboration between organisational units, nurse managers underlined the importance of enabling nurses to rotate between roles in their organisations, as they saw this as an important way of becoming familiar with further career opportunities.

“*We have different staff in pre-hospital care and in the emergency department and both are developing their operations– we should break boundaries to exploit (others′ work for career development).”* (Participant 3; Registered nurse)

#### 3.2.2. Clear and Attainable Career Model in the Organisation

*Competence-based role alignment* across an organisation was emphasised as a central mechanism for career planning and development. To this end, participants are suggesting using a voluntary register in which nurses can document their ambitions, training and competencies, providing information which the employer could then use when looking for a suitable employee. It was suggested that this would require a dedicated coordinator in each organisation. Participants also mentioned career coaching as a possible way of focusing nurses’ competence development, possibly using coaches from outside the organisation. Participants also mentioned tailoring job descriptions, considering career planning and development so that employees can pursue and/or utilise training and new skills in their work.

*“Head nurses, nurse managers should have some lists of people′s competencies and interests. We could look [to put the] right people in the right places [in our] own organisation* … *These projects and stuff revolve around such a small group of people. The know-how and willingness can be found elsewhere too.”* (Participant 18; Registered nurse)

Participants identified a need for standardised *career ladders* and called for the use of clear job and role descriptions in the organisation to enable equal, transparent, and systematic career planning and development. They saw career ladders as important for determining competence requirements and expectations, for example, helping nurse managers with shift planning and enabling nurses to set career goals. Nurses brought up the need for a shared national career model. Nurse managers identified a need to modify organisation‐wide models to accommodate the unique features of particular units.

*“In my workplace it has been said that there needs to be a person responsible for diabetes care. But there is no description for what it means and what competencies this person should manage* … *This is crazy, no one knows what is expected.”* (Participant 19; Nurse manager)

A central topic in the interviews concerned *transparent recognition and rewards* of nurses’ career development, around which participants felt there were significant deficiencies in the field of nursing. They said that nurses get enhanced assignments from physicians when they acquire additional education and qualifications, but do not receive financial compensation, as their careers progress, many nurses move from shift work to daytime work. However, as a component of nursing salaries is intended to compensate for working shifts, career progression can actually cause a nurse’s income to drop. They also discussed differences in work performance and said that high‐quality work should be rewarded. Nurses felt that they should be rewarded for remaining in nursing over the long term. Participants also highlighted that people need positive verbal feedback and recognition for their efforts, in addition to financial compensation.

#### 3.2.3. Daily Career Support and Follow‐Up

Participants identified the need for nurses to have *individual career planning*, and nurse managers said that these work as tools to promote career progression. For example, they enable them to suggest suitable trainings and opportunities to participate in developmental work. Individuals’ plans could also help with workforce anticipation and filling vacancies with the most suitable professionals within the organisation. Individual career plans are also valued as a tool for assessing career progress.

Participants emphasised that nurse managers have a key role in providing daily support to nurses’ career planning and development and assigning staff with suitable tasks*, using reflections and performance reviews*. These enable nurse managers to understand a nurse’s career aspirations, have open and reflective discussions with them, and provide feedback and encouragement. Participants noted that nurse managers are in an optimal position to recognise nurses’ interests, wishes, and potential and to spur them into taking steps towards new roles or deepening their competence. Annual one‐to‐one development discussions provide a concrete opportunity for nurses and nurse managers to discuss career progression. Nurse managers also influence nurses’ working arrangements and employment and can thus enable activities such as further study. They are also in a position to assess nurses’ performance and thus identify individuals who may deserve a higher rate of pay.

*“I have had a good manager who has noticed my restlessness and she has supported me at that point (to develop own career).”* (Participant 18; Registered nurse, worked in different places)

Nurse managers mentioned *peer support and mentoring* as supports to individuals’ career planning and development. These are provided by more experienced colleagues and involve transferring tacit knowledge, exchanging experiences, clearing things up, discussing ethical dilemmas, and finding solutions together. Trust was felt to be central to these practices and, compared with discussions with a nurse manager, for example, might make it easier for an individual to express their own thoughts. Overall, the work community was emphasised as significant to one’s career aspirations. Some described the value of colleagues’ role modelling, inspiring, and encouraging discussions and feedback in helping them to identify their own strengths and take steps towards career development. At the same time, some had encountered envy and being undervalued and negative attitudes towards further study or role progression. Some had even hidden their degrees from others because they feared inappropriate treatment in the work community. With regards to peer support, nurse managers also highlighted the importance of new employees being well treated and familiarised with the work community.

*“Mentoring is peer support, so a nurse manager cannot mentor an employee*… *It would be really important that every level, in our world I mean nurses, nurse managers and chief nursing officers, that every level, every professional group has its own network of mentors.”* (Participant 9; Nurse manager)

According to the participants, it is important that there is systematic *career development evaluation*. They suggested various direct and indirect indicators that could be used for this. Individual career plans could be scrutinised at specified intervals, and assessment could focus on pursuing planned training and study leaves and achieving degrees and job description changes. Information about progress could be gathered through discussions and staff surveys, and indirect indicators of progress could include employee satisfaction, coping, absenteeism, workforce retention, and quality of care.

*“Employees should probably be interviewed [about] how this (career planning and development) has been presented in the workplace and how has it gone* … *Employees′ voice should be heard [so as not] to make assumptions at the administrative level.”* (Participant 5; Registered nurse)

## 4. Discussion

Based on our findings, career planning and development emerge as a goal‐oriented professional strategy which is based on nurses’ professional rights. It manifests as competence proliferation and advanced roles and strengthens nurses’ professional power. Organisational support for nurses’ career planning and development requires a multilayered, progressive structure which is reflected in career‐oriented organisational values, a clear and achievable career model, and daily support and follow‐up. In our study, nurses reported that limited career opportunities and insufficient support for professional development pose a threat to the meaningfulness of nurses’ working lives. The results also revealed that bedside nursing is under, despite the critical need for more nurses to pursue and remain in clinical practice involving direct patient care.

Our findings deepened previous knowledge about the significance of horizontal career support in the workplace and nursing communities [10, 22]. In particular, our findings highlight a strong desire for mentoring, which is felt beneficial by nurses. Mentoring by more experienced colleagues supports equal and open reflection [10], facilitates the internalising of new duties [36] and helps manage transition processes that are often so psychically burdensome [10]. This suggests that structured peer mentoring is a key element in building a career supportive work culture. A systematic mentorship framework would benefit both mentors and mentees by increasing commitment and fostering career‐oriented work culture. Additionally, our findings also highlighted the importance of an established work culture where career development intentions are discussed regularly in meetings and actively promoted with more or less official recognitions and award.

Our findings highlighted nurse managers’ vantage point to coordinate staff into tasks that match their competencies and interests. The results elaborated previous knowledge of nurse manager’s key role in enabling and supporting nurses’ career planning and development [25, 37] by sharing information about career opportunities [38] and training [37]. In this study, a great emphasis regarding their role in career planning and development was given to the need for personal social support, which is also known to contribute employee’s career planning attitudes and decisiveness [4]. This study also found that discussions and reflection with a nurse manager were found crucial to nurses’ career planning. This discussion can be included as a natural part of annual performance reviews. Based on our results, the aim of the discussions should be to identify one’s true interests but also to boost and encourage them to take new challenges in their working life.

Our findings provide new insights into the role of nurse managers in facilitating nurses’ career development. Based on our study, when frontline nurse managers supported nurses’ career plans, they often focused their encouragement on opportunities within their own unit. Although well‐intentioned, this limited focus resulted in frustration among nurses and, in some cases, increased their intention to leave the profession. This highlights the critical need for nurse managers to adopt a broader perspective—one that considers both organisational and profession‐wide career pathways. Supporting nurses career planning should not be limited to tasks at the unit level but should be aligned with strategic goals across the organisation and the wider healthcare system. Thus, to ensure both career progression and nurse retention, the first and most urgent step is to develop a strategic plan and clear evaluation criteria for a sustainable workforce framework. That framework should address not only organisational needs but also the broader health service levels. To achieve that goal, nurse managers must have the necessary skills [16] and be committed to sustainable nursing workforce planning across sector.

Our research findings highlight the need to further develop clearer career path models at both the organisational level and the national level. In addition to meaningful, competence‐based tasks and responsibilities, adequate and progressive reward structures are essential for supporting career development. However, internationally, poor staffing levels in daily work and a challenging economic situation in healthcare pose a risk to nurses’ participation in continuing training and could potentially slow or halt their career progression [9, 37]. Furthermore, the lack of salary progression together, with the fact that further training and competence development often have no impact on nurses’ pay, poses a serious risk of nurses leaving the profession. This underscores the importance of aligning career development with financial incentives so that nurses feel that their efforts and growth are valued. In the future, more research is needed to evaluate the effect of balancing improved salary structures with the economic consequences of nurses leaving the profession. This information is required for short‐ and long‐term strategic planning at both organisational and national levels to support nursing management in nurses’ career planning and development and ensure sustainable workforce planning in healthcare.

Although this study was situated within the Finnish healthcare system, the identified needs for structured career planning, strong managerial support, and transparent career models are consistent with international literature. Therefore, the findings may be transferable to other healthcare systems with similar organisational structures and workforce challenges, particularly in countries aiming to enhance nurse retention and professional development.

### 4.1. Limitations

The limitations of this qualitative study relate to the target group, data collection and analysis of the data. As the participants were from one hospital district and primary healthcare services, which means that the findings should be transferred with caution to healthcare and career contexts beyond Finland. As our aim was to understand the shared phenomenon within the organisation, and as the number of voluntary participants was limited, we included all individuals who met the inclusion criteria. However, we enhanced the representative sample of nurses and nurse managers, but applying more detailed analysis of maximum variation sampling could have increased the diversity of perspectives. However, this study provided inductive knowledge that contributed to previous international literature, enabling the development of an instrument to measure the phenomenon in different contexts. During data collection, participants may have provided socially acceptable responses [39]; however, they were encouraged to share all insights openly and without judgement during the interviews. During the analysis phase, there is a risk that the researchers may have misinterpreted the participants’ intended meanings. We did not conduct follow‐up interviews or return the transcripts to participants, as we aimed to avoid burdening nurses during their busy workdays. To minimise misunderstandings, the data were analysed collaboratively within a multiagency research team, and during discussions, the data were considered understandable.

## 5. Conclusions

The career planning and development of nurses is important for the quality and efficiency of services, as well as for the attractiveness of nursing as a profession. Career development should support nurses’ professional commitment, motivation and retention within the profession. Although nursing encompasses various career paths, recognised career advancement within direct patient care remains limited. This is a particularly notable finding, given that bedside nursing represents the core of the profession, and the majority of the nursing workforce should be able to pursue meaningful and progressive careers in this area. Based on our results, it is recommended to implement a systematic strategy for peer mentoring and encouraging open and regular discussions about career opportunities as a part of career‐oriented work culture. These discussions could be an integrated part of annual performance reviews, ensuring continuity and visibility of career planning. Importantly, nurses’ career opportunities should not be restricted to the unit level. Nurse managers play a key role in developing and actively promoting career paths at both organisational and broader professional levels. Strategic frameworks at healthcare county or system level, considering continuing training and reward structures, are needed to support sustainable workforce planning and retention of nurses in the profession. However, further research is needed to identify effective and supportive career ladder models. It is also essential to ensure that nurse managers have the necessary competencies and understanding to support nurses’ career planning and development in a sustainable way.

## Funding

This work was supported by the “The Wellbeing Services County of Satakunta” (388/2022), Pori, Finland. Open access publishing was facilitated by the Turun Yliopisto, as part of the Wiley‐FinELib agreement.

## Conflicts of Interest

The authors declare no conflicts of interest.

## Supporting Information

Additional supporting information can be found online in the Supporting Information section.

## Supporting information


**Supporting Information 1** Supporting Table 1. The supporting document provides a structured analysis of the study followed the Consolidated Criteria for Reporting Qualitative Research (COREQ) checklist.

**Supporting Information 2** Supporting Table 2. The supporting document provides a description of literature‐based, semistructured interview guide in this study.

## Data Availability

Research data are not shared.
