# Supplementary material for: Nursing Professionals’ Perceptions of Career Planning and Development in Nursing and Organisational Support
Source: J Nurs Manag. 2026 Mar 18;2026:8843697. doi: 10.1155/jonm/8843697 (PMC13140928; doi:10.1155/jonm/8843697)
Supplement: Supplementary file 2 — Supporting Information 2 Supporting Table 2. The supporting document provides a description of literature‐based, semistructured interview guide in this study. [file JONM-2026-8843697-s002.docx]

Supplementary table 1. The interview guide.

| **Theme 1** | **Career and career planning in nursing** | |
| --- | --- | --- |
|  | •What do you think career and career planning in nursing means? | |
|  | •What are or could be nurses' career options? | |
|  | •How do nurses proceed in their career, what is career development? | |
|  | •What are the special features of a career and career plan in nursing? | |
|  | •What does a good career look like? | |
| **Theme 2** | **Career and career planning in nursing education and working life** | |
|  | •How were career paths and planning presented in your education? | |
|  | •How are career paths and planning addressed in your working life? | |
| **Theme 3** | **Meaning of career planning in nursing: employee, group of professionals, organisation, society** | |
|  | •Why is career development needed, why not? | |
|  | •What is the goal of career planning? | |
|  | •How can the success of career planning be evaluated from the perspective of an individual employee or an organisation? | |
|  | •What can result from not promoting career planning in nursing? | |
| **Theme 4** | **Means and implementation of career planning for nurses** | |
|  | •What is nurse's own role in career planning? | |
|  | •How can career planning be promoted in the organisation? | |
|  | •How can nurse managers promote career planning? | |
|  | •What should be taken into account at the societal level in relation career planning by care workers? | |
| **Ending discussion** | | What else would you like to say about careers and career planning in nursing? |
